# Conditions Enabling the Persistence of Cooperating Synthetase, Ligase, and Mutation-Inhibitor Catalytic Polymers

**DOI:** 10.1007/s00239-026-10323-6

**Published:** 2026-06-09

**Authors:** Zhen Peng, Alex M. Plum, Rahul Kartha, Emily M. Jacobson, David A. Baum

**Affiliations:** 1 Wisconsin Institute for Discovery, University of Wisconsin–Madison, Madison, WI 53706 USA; 2 State Key Laboratory of Bioreactor Engineering, School of Biotechnology, East China University of Science and Technology, Shanghai, 200237 China; 3 Department of Botany, University of Wisconsin–Madison, Madison, WI 53706 USA; 4 Present Address: Department of Physics, University of California San Diego, La Jolla, CA 92093 USA; 5 Present Address: Meta Platforms, Inc., 1 Meta Way, Menlo Park, CA 94025 USA

## Abstract

In origins-of-life research, a key challenge is to explain the emergence of polymers of sufficient length to confer the complex functions needed for genetic inheritance. Previous studies have demonstrated that prebiotic environments enabling multilevel selection can facilitate the survival of cooperating polymers such as ribozymes, which allows that complex functions undertaken today by long polymers might have originated from multiple simpler functions undertaken by shorter polymers. To further investigate this possibility, we developed a new computational model of cooperative catalytic and replicating polymer systems that avoids tracking all possible polymer sequences. This approach scales well computationally, avoiding the need to set an a priori cap on polymer length. We first validated this model by replicating key conclusions of previous studies, for example that the persistence of cooperative synthetase-ligase systems is facilitated by both intrinsic factors (shorter length, higher catalytic efficiency) and by factors that promote multilevel-selection (compartmentalization, slower diffusion). We then explored the effects of introducing a mutation inhibitor into a cooperative synthetase-ligase system. The results support the possibility that mutation inhibition could have arisen, not through the appearance of a single proofreading polymerase, but through the emergence of distinct, mutation inhibiting catalysts.

**Supplementary Information:**

The online version contains supplementary material available at 10.1007/s00239-026-10323-6.

## Keywords

Computational modeling, Error threshold, Molecular cooperation, Multilevel selection, Origin of life, RNA World

**Supplementary Information:**

The online version contains supplementary material available at 10.1007/s00239-026-10323-6.

## Introduction

In origins-of-life research, it has long been appreciated that the initial “RNA World” – if it ever existed – was likely composed of multiple cooperating, short ribozyme species rather than a single RNA-dependent RNA polymerase (RDRP) (Eigen and Schuster 1977, 1978a, b; Hordijk and Steel 2013; Czárán et al. 2015; Kim and Higgs 2016; Szilágyi et al. 2020; Adamski et al. 2020). Minimally, the first functional polymerases likely cooperated with ribozymes that catalyzed metabolism-like functions, such as monomer-synthesis or monomer-activation. Prior work has shown that systems of cooperating ribozymes can become established when spatial structure exists and results in multilevel selection (Szathmáry and Demeter 1987; Takeuchi and Hogeweg 2009; Branciamore et al. 2009). Some models have imagined the spontaneous appearance of protocells (Szathmáry and Demeter 1987; Ma et al. 2010a; Ma and Hu 2012), while others have considered systems that are organized on an abstract mineral surface with limited diffusion (Szabó et al. 2002; Branciamore et al. 2009; Szilágyi et al. 2020). In both cases, spatial structure can allow cooperating ribozymes to persist and escape parasitic RNAs.

Almost all prior models of an RNA world have focused on RDRP ribozymes, whose functionality is guided by modern processive polymerases. These are complex, multi-functional catalysts that achieve RNA synthesis in two potentially distinct steps: (1) promoting the formation of a phosphodiester bond between the 3’-end of a non-template strand and the 5’-end of a free nucleotide, and (2) relocating to the 3’-end of the newly incorporated nucleotide. There are good reasons, however, to assume that the first template-guided RNA synthesis was catalyzed by ligases rather than polymerases. First, ligase activity is much simpler than polymerase activity because processivity is not required (Ma et al. 2010b). Second, ligation can happen between oligomers, which would likely have existed alongside the monomers and full templates that are used by polymerases. Third, experimental studies have detected ligase or ligase-like activity in relatively short ribozymes (Bartel and Szostak 1993; Ekland and Bartel 1995; Jaeger et al. 1999; Paul and Joyce 2002; Attwater et al. 2018; Nomura and Yokobayashi 2019; DasGupta et al. 2024; Biswas and DasGupta 2026; Gianni et al. 2026). Thus, it is important to evaluate models where ligases rather than polymerases are responsible for catalyzed, template-guided replication, and see if viable, cooperative systems can emerge.

A key consideration in all RNA-world models is the mutation rate. The fidelity of RNA replication needs to be high enough, relative to the length of functional ribozymes, to overcome Eigen’s error-threshold (Eigen 1971). This principle argues that the earliest catalytic polymers would have been short, making it improbable that evolution began with an RDRP ribozyme resembling modern polymerases, which have catalytic domains that enable error correction. One resolution is to envisage the spontaneous appearance of short, error-prone polymerases that eventually gave rise to longer descendants with lower mutation rates (Szabó et al. 2002; Shay et al. 2015; Shah et al. 2019). An alternative, which we explore here, is the appearance of distinct classes of cooperating ribozymes that serve as mutation inhibitors. For example, a ribozyme could lower the effective mutation rate by manifesting exonuclease-like activity, by detoxifying mutagens, or by reducing the concentration of non-canonical “wildcard” nucleotides (e.g., inosine).

In this study, we modeled systems of cooperating polymers that include *synthetases*, which synthesize activated monomers from precursor food, *ligases*, which foster template-mediated ligation and, thus, replication of template sequences, and *mutation inhibitors*, which act to reduce the probability of base misincorporation. To model such a system, we needed an approach that could handle any polymer sequences that emerge, regardless of length. Ligase-catalyzed replication will frequently generate truncated or elongated sequences, which need to be tracked since they can act as parasites (non-functional sequences that can serve as templates) or be extended/hydrolyzed later to generate functional sequences. However, handling explicit sequences in numerical simulations is computationally challenging due to the combinatorial explosion that arises when tracking explicit polymer sequences.

To address this computational challenge, we developed a spatially-structured stochastic model that is inspired by the theory of surface metabolism (Wächtershäuser 1988, 1997) and draws on aspects of the stochastic corrector model (Szathmáry and Demeter 1987; Grey et al. 1995; Zintzaras et al. 2002) and the model of metabolically coupled replicator systems (Czárán et al. 2015; Kim and Higgs 2016). Using a strategy resembling the computer game Tetris®, the computational load can be set, not by the number of possible polymer sequences, but by the size of compartments (ecosystems) and, to a lesser extent, by the count of realized catalytic polymer molecules per compartment, and the number of compartments. This approach circumvents the combinatorial explosion problem, making it possible to run simulations without needing to define a maximum polymer length. We decided to model an abstract polymer system with just two monomer types, rather than RNA per se, given the possibility that alternative types of polymers were involved in templated-mediate replication during origins of life, for example peptide nucleic acids (Nelson et al. 2000; Nielsen 2007; Piette and Heddle 2020), threose nucleic acids (Schöning et al. 2000; Wang et al. 2022; Wang and Yu 2024), or peptide amyloids (Maury 2018; Greenwald et al. 2018).

Using this model, we first replicated key results from previous studies that investigated the cooperation of polymerases and catalysts playing metabolic roles, such as synthesis of monomers (e.g., nucleotides) (Szabó et al. 2002; Shay et al. 2015; Czárán et al. 2015; Könnyű et al. 2015; Kim and Higgs 2016; Shah et al. 2019; Szilágyi et al. 2020). We corroborated the finding that discrete compartments with spatially-structured diffusion promote cooperation relative to well-mixed systems (Czárán et al. 2015; Kim and Higgs 2016). We also confirmed that higher catalytic efficiency is associated with higher viability and that greater polymer length is associated with lower viability (Szabó et al. 2002; Shay et al. 2015; Czárán et al. 2015; Könnyű et al. 2015; Kim and Higgs 2016; Shah et al. 2019; Szilágyi et al. 2020). Then, having established the validity of the model, we added a mutation inhibitor and examined the conditions needed for the persistence of synthetase, ligase, and mutation inhibitor polymers. We found evidence that the length threshold for the persistence of cooperating catalytic polymers can be elevated by the presence of mutation inhibitors. Together, these results support the idea that the first template-mediated replication system arose in contexts promoting multilevel selection and consisted of multiple short polymers instead of long, multi-functional polymers like proofreading polymerases.

## Results

### A Simplified Spatial Model to Efficiently Simulate Prebiotic Genetic-Catalytic Polymers

We set out to explore the ability of cooperative polymer systems to survive and evolve in a prebiotic environment using an abstract model that captures the effects of diverse physicochemical factors. A core challenge is the combinatorial explosion that most RNA-world models confront. Even if template-guided polymerization is treated as a single reaction (as in replicator equations (Schuster and Sigmund 1983; Sigmund 1986; Stadler 1991)), and only two monomer species are considered, the computational load increases exponentially with the maximum polymer length allowed. For example, for a maximum length of 20, there are more than 10^6^ single-stranded polymer species that need to be tracked. Moreover, if (partially) double-stranded polymers and the complexes formed by replicases, templates, and nascent strands are considered, then the number of tracked species would be orders of magnitude higher still. Additionally, if we allow for random breakage of polymers and ligation among fragments of any length, which could be realistic in prebiotic environments, then the number of tracked reactions would quickly become infeasible. To solve this challenge, we adopted an adsorption-based strategy, which assumes that catalyzed reactions only occur to molecules adsorbed to a mineral surface or to other adsorbed molecules, such that the number of chemical bonds that may be formed or broken is limited by the size of mineral surface. With such a model, we do not need to track all possible polymer sequences but simply update the status of a limited number of bonds during a reaction cycle and then update realized polymer sequences based on changes in bond status between cycles.

Another challenge is to model polymers capable of acting independently to inhibit mutations. Previous approaches have assigned mutation inhibition to polymerases themselves, such that each polymerase sequence has an intrinsic error rate. We sought to model mutation inhibition by an independent polymer species where the realized mutation rate varies with the concentration of the mutation-inhibiting polymer. To achieve this, we considered the possibility of a monomer being chemically modified into a “wildcard,” which does not reliably pair with its correct complementary base. This allows us to consider mutation inhibitor catalysts, which are polymers that lower the probability of normal monomers becoming wildcards. Multiple physicochemical mechanisms could be responsible for mutation inhibition, such as correcting wildcards back to normal monomers or decomposing environmental mutagens that generate wildcards. This normal-monomer-versus-wildcard system provides a simple way to simulate replication, mismatch-induced mutations, and mutation inhibition.

We developed a multi-reactor computational model that significantly simplifies the process of simulating polymer systems and avoids the need to pre-specify maximum sequence length (see Sect. “Methods”). Three types of functional polymers are considered: a “synthetase” which catalyzes the formation of activated monomers from food, a “ligase” which links activated monomers or polymers to other monomers or already formed polymers, and a “mutation inhibitor” which reduces of probability of mutation. Here, the catalysts (at least synthetase and ligase) cooperate, providing service to the entire polymer system, but also incur a cost insofar as a sequence acting as a catalyst cannot also act as a template.

**Fig. 1:**
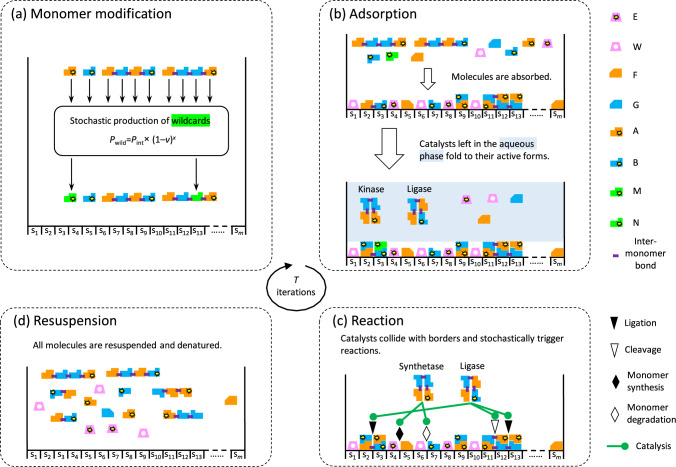
Four phases of the model. Each reactor has *m* sites. Each site can adsorb a food molecule, a waste molecule, a free monomer, or an incorporated monomer. Energy food (E) and waste (W) are non-directional along the horizontal dimension, whereas structural foods (F and G), free monomers (A and B), wildcards (M and N), and polymers are directional. **a** Phase 1: Monomer modification. Free and incorporated monomers stochastically become “wildcards,” which are monomers that can pair with any type of monomer, with a probability *P*_wild_ equal to an intrinsic wildcard-production probability *P*_int_ multiplied by (1–*v*)^*x*^ where *x* and *v* are the quantity and efficiency of mutation inhibitors, respectively. **b** Phase 2: Adsorption. Molecules are randomly adsorbed to the surface until no more adsorption can occur. When a directional molecule, such as a free monomer or a polymer, is adsorbed to the surface as a first-layer adsorbate, it stochastically makes its adjacent sites inaccessible to other directional molecules. This reduces the occurrence of fusion between reverse complements of different first-layer templates and, thus, can control the rate of fusion-induced mutations in the second layer. At the end of this phase, all wildcard states are erased. **c** Phase 3: Reaction. Each catalyst in the aqueous phase randomly collides with borders between adjacent surface sites *N* times, where *N* is drawn from a Poisson distribution. Once a catalyst collides with a reactive border, it has a chance to cause a reaction. **d** Phase 4: Resuspension. All adsorbed molecules are released into the aqueous phase

This model assumes that polymer systems iterate through four phases. In Phase 1 (monomer modification), free and incorporated monomers (A, B) stochastically become “wildcards” (M, N) with a probability equal to an intrinsic wildcard-production probability multiplied by a function that is negatively associated with the number and efficiency of mutation inhibitors (Fig. 1a). A higher number of monomers entering the next phase as wildcards means a higher rate of mismatch-induced mutations. In Phase 2 (adsorption), molecules of all types are adsorbed to a mineral surface or on top of a lower layer of adsorbed molecules until no more adsorption can occur, similar to the way that Tetris® bricks fall on other bricks (Fig. 1b). Then all wildcard states are erased by replacing M and N with A and B, respectively. This mimics real mismatch-induced mutations in that the template strand (i.e., the first-layer polymer) is not modified but the strand that will be produced (by ligating the second-layer free or incorporated monomers) can imperfectly match the template and incorporate mutations. Certain combinations of molecules occupying adjacent sites are “reactive borders” (Fig. 1c) where a bond may be formed or broken in the next phase. In Phase 3 (reaction), catalytic polymers that were not adsorbed are allowed to randomly collide with borders. Whenever a catalytic polymer (e.g., ligase) collides with a reactive border, it has a probability, its *catalytic efficiency*, of causing a reaction to occur (Fig. 1c). In Phase 4 (resuspension), all adsorbed molecules are released (Fig. 1d). Given that at least some synthetase and ligase polymers exist at the beginning, iterating these four phases for multiple cycles is conceptually similar to iterating the steps of annealing, extension, and denaturation in the polymerase chain reaction (PCR). Multiple such cycles form a generation (Fig. 1).

**Fig. 2:**
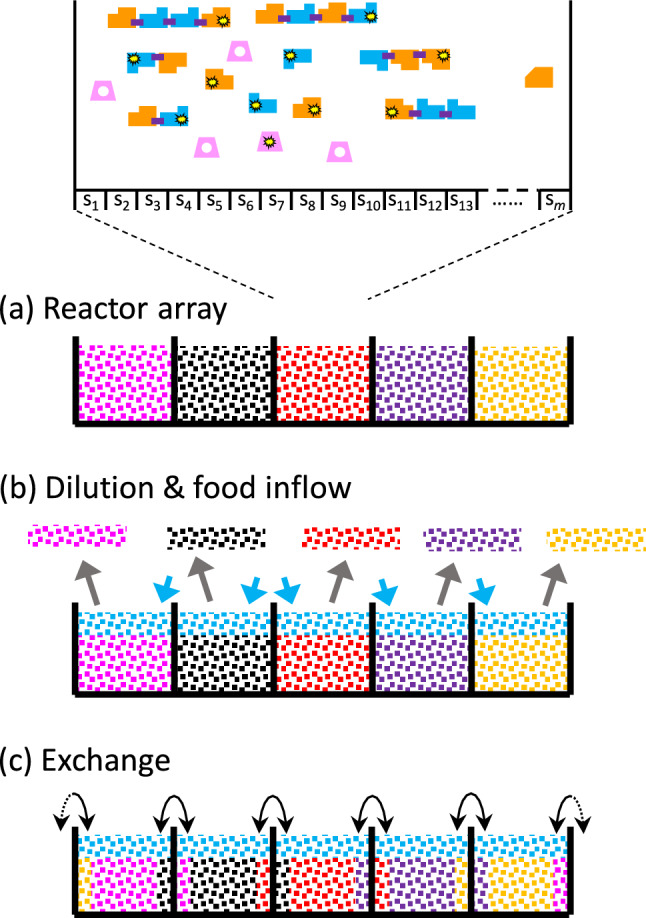
A reactor array. **a** Multiple reactors can form a circular array, where the rightmost and the leftmost reactors are neighbors. **b** Reactors have a portion of the contents (grey arrows) replaced by foods (cyan arrows). **c** Reactors exchange their contents. This example shows the neighborhood-dispersal regime; there may also be other schemes; see Sect. “Externally Provided Compartments and Slow Inter-Compartment Exchange Help Cooperating Polymers Persist” and Fig. 3

Simulations were conducted for multiple reactors through multiple generations in order to allow for multilevel selection, which could aid in the enrichment of cooperating polymers (Szathmáry and Demeter 1987; Hogeweg and Takeuchi 2003; Takeuchi and Hogeweg 2009). We created a circular array of reactors (the leftmost and rightmost reactors in Fig. 2a are also neighbors). After a generation ends, a fraction of each reactor’s contents are replaced by food from an external source (Fig. 2b), where the proportion of the contents that is replaced each generation is defined as the *dilution rate*. Then reactors exchange a portion of their contents, representing overflow or diffusion (Fig. 2c). More details of our model can be found in Sect. “Methods”.

To demonstrate the ability of this model to handle long polymers, we initiated the reaction system with only food species (E, F, and G), running the simulations with high levels of spontaneous activation/ligation and an elevated probability of spontaneous fusion (see Sect. “Methods”). We ran 30 replicates, each with a single reactor of 200 sites (i.e., 199 borders), for 20 generations. We found that the longest polymers in the final populations have an average length of ~ 44 (Table S1) and the longest polymer among all replicate simulations is a 63-mer (Table S1, Replicate 3). Running all the 30 replicates in parallel took approximately 29 hours using the high throughput computing service at the Center of High Throughput Computing at the University of Wisconsin–Madison (Center for High Throughput Computing 2006).

### Replicating Previous Findings to Test the Validity of Our Model

In order to assess the validity of our model, we sought to replicate, at least qualitatively, prior findings (Szabó et al. 2002; Shay et al. 2015; Czárán et al. 2015; Könnyű et al. 2015; Kim and Higgs 2016; Shah et al. 2019; Szilágyi et al. 2020). In this section, there is no mutation inhibitor, and the intrinsic wildcard production probability was set to *P*_int_ = 10^–6^.

#### Externally Provided Compartments and Slow Inter-Compartment Exchange Help Cooperating Polymers Persist

In accordance with classical research on the evolution of cooperation driven by group selection (Smith 1964), it was found that externally provided spatial structure, which can be envisaged as pores in a rock surface, combined with limited inter-compartment exchange, could promote cooperation and suppress cheating (Czárán et al. 2015; Kim and Higgs 2016). We evaluated a scenario in which synthesis of polymers requires the presence of both a synthetase polymer that promotes the activation of monomers and a ligase polymer that converts monomers into polymers in a template-guided manner. We evaluated the probability that both the synthetase and ligase functions would persist if they happened to both arise spontaneously in a single reactor using three groups of simulations.

**Fig. 3:**
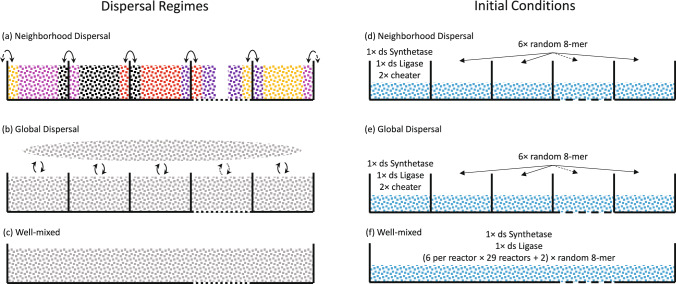
Three dispersal regimes and their initial conditions. **a** In the neighborhood-dispersal regime, a randomly selected 60% of a reactor’s contents (by molecular count) remain in the same reactor, 20% move to the left reactor, and 20% move to the right reactor. **b** In the global-dispersal regime, a randomly selected 60% of a reactor’s contents remain in the same reactor and 40% contribute to a shared pool that is then randomly redistributed among all the reactors. **c** In the well-mixed regime, there is only one giant well-mixed reactor. **d**, **e** Simulations for the neighborhood-dispersal and global-dispersal groups are initialized with one 8-mer synthetase (ABAABABB) and its reverse complement, one 8-mer ligase (BBAAABAB) and its reverse complement, and two random 8-mer cheater polymers in the first reactor, whereas the other 29 reactors are seeded with six random 8-mers. **f** Simulations for the well-mixed group are initialized with the same sequences as the other two conditions, but all in a single reactor

In the first group, an array of 30 reactors (each with 100 sites) experience *neighborhood-dispersal*, where a portion of each reactor’s volume is exchanged with its two neighbors (Fig. 3a). In the second group, 30 reactors (each with 100 sites) experience *global-dispersal*, where a fraction of reactor contents are randomly redistributed among all reactors (Fig. 3b), similar to Maynard Smith’s haystack model (Smith 1964). In the third group, there was a single *well-mixed* reactor, with 3000 sites (Fig. 3c).

All simulations used low spontaneous reaction rates and low probabilities of fusion-induced and mismatch-induced mutations (see Sect. “Methods”). The neighborhood-dispersal and global-dispersal groups were initialized with one reactor seeded with one 8-mer synthetase (ABAABABB) and its reverse complement, one 8-mer ligase (BBAAABAB) and its reverse complement, and two random 8-mers, while the other 29 reactors were each seeded with six random 8-mers. All reactors began with their food concentrations the same as the external food source (Fig. 3d–e). The well-mixed group included the same 180 sequences (6 polymers times 30 reactors) all in the single reactor (Fig. 3f).

For each group, multiple simulations were performed, which allowed us to estimate the proportion of simulations where both cooperators persisted. We defined persistence as there being, after 50 generations, at least one reactor where the synthetase, the ligase, and their reverse complements were all present.

Although we showed in Sect. “A Simplified Spatial Model to Efficiently Simulate Prebiotic Genetic-Catalytic Polymers” that our model can handle polymers longer than 60-mers, we chose to focus initially on 8-mer catalysts. We do not need long functional polymers to assess the relative effects of different factors since, even with 8-mers, there are many more non-functional cheater sequences ((2^2^ + 2^3^ + … + 2^8^) – 4 = 504) than functional sequences (2) and reverse complements of functional sequences (2). By using sequences of this length, computation is more efficient, allowing us to explore many different factors within a reasonable timeframe.

**Fig. 4:**
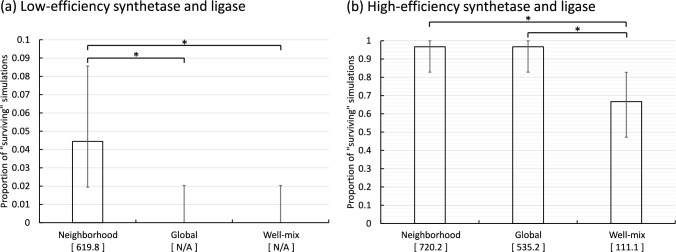
Compartmentalization and slow inter-reactor exchange help cooperating polymers survive. Graphs show the proportion of simulations where both synthetase and ligase sequences survived. Error bars show the 95% Clopper-Pearson confidence intervals. The number in brackets below a column indicates the average count of catalytic sequences or their reverse complements per surviving simulation. **a** Each column is based on 180 replicate simulations; synthetase efficiency = 0.0005, ligase efficiency = 0.02. **b** Each column is based on 30 replicate simulations; synthetase efficiency = 0.005, ligase efficiency = 0.2. Note that **a** and **b** have different vertical axis scales. For all simulations, the dilution rate was 0.05; external food counts per unit volume were 1000 for E, 500 for F, and 500 for G. Significant differences are indicated by stars

As shown in Fig. 4, the probability of persistence is sensitive to catalytic efficiency, and is enhanced in the neighborhood- and global-dispersal groups, suggesting a role for multilevel selection. When catalytic efficiencies are low (synthetase efficiency = 0.0005, ligase efficiency = 0.02), the cooperating catalysts are only ever viable in the neighborhood-dispersal group (Fig. 4a). In most cases where persistence did not occur, extinction occurred in the first few generations. This helps explain the relative success of the neighborhood-dispersal model, since copies of the functional species produced in the first generation only spread out to its two neighbors, increasing the chance that one or both neighbors will have both cooperating catalysts in the second generation. When catalytic efficiencies are high (synthetase efficiency = 0.005, ligase efficiency = 0.2), catalysts could persist in all three cases (Fig. 4b), but even here the persistence probability for well-mixed group was significantly lower than the other two.

Since the neighborhood-dispersal model is geologically plausible and seems to be the most effective organization for enabling the persistence of cooperating polymers in this case, we utilized this model for all subsequent analyses.

#### Systems Consisting of Polymers with Higher Catalytic Efficiencies are More Likely to Survive

**Fig. 5:**
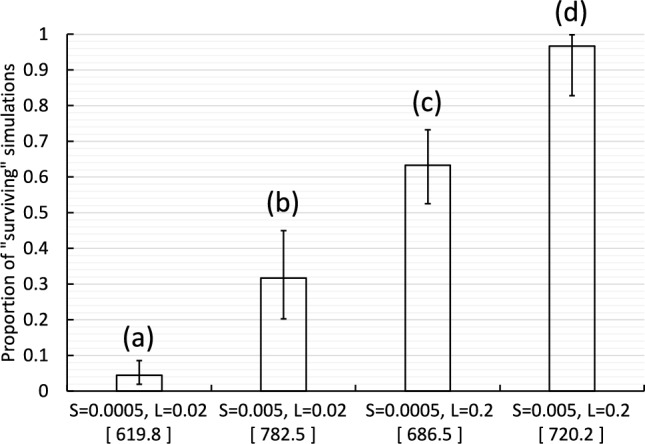
Catalytic efficiencies of both functions are positively correlated to viability. Error bars show the 95% Clopper-Pearson confidence intervals (all columns are significantly different). The bracketed number below a column indicates the average count of catalytic sequences or their reverse complements per surviving simulation. **a** Synthetase efficiency = 0.0005, ligase efficiency = 0.02, 180 replicates. **b** Synthetase efficiency = 0.005, ligase efficiency = 0.02, 60 replicates.** c** Synthetase efficiency = 0.0005, ligase efficiency = 0.2, 90 replicates. **d** Synthetase efficiency = 0.005, ligase efficiency = 0.2, 30 replicates. Except as indicated, initial conditions and model parameters are the same as Fig. 4

The data summarized in Fig. 4 already showed that elevating the catalytic efficiency of both the synthetase and ligase polymers increases the probability of persistence. We wondered whether a similar benefit might be seen if just one of the functional catalysts is more efficient as suggested in previous publications (Szabó et al. 2002; Shay et al. 2015; Shah et al. 2019), because this might enable ratchetting up of system viability, one function at a time. Indeed, simulations using different combinations of high- or low-efficiency synthetase and high- or low-efficiency ligase (Fig. 5) show that enhancing either catalytic efficiency ten-fold significantly enhances the probability of both cooperators persisting. This follows because higher catalytic efficiencies mean that more food is converted into polymers within a generation (Fig. 1), which lowers the probability that catalytic polymers (of both kinds) are lost during inter-generation dilution (Fig. 2).

#### Systems Consisting of Shorter Polymers are More Likely to Survive

Longer polymers have greater potential to adopt diverse secondary and tertiary structures, opening up more potential catalytic roles and raising the maximum plausible catalytic efficiency. For example, long polymers have more potential for conformational change, which can greatly increase catalytic efficiency (Rivoire 2024). However, the rate at which longer functional polymers can be produced is lowered by the fact that their production consumes more resources and requires more reactions. Additionally, if mutations are frequent, longer templates will tend to yield more non-functional products. As a result, the viability of ribozyme systems is greater when functional polymers are shorter (Szabó et al. 2002; Könnyű et al. 2015; Szilágyi et al. 2020).

**Fig. 6:**
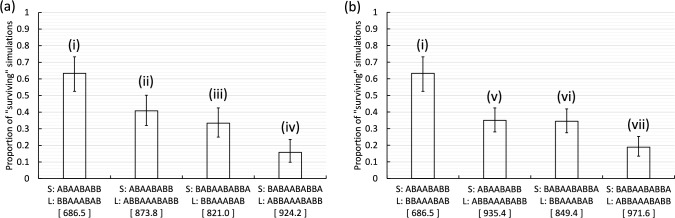
Catalyst length is negatively correlated to viabilities of polymer systems. Initial conditions and model parameters are the same as Fig. 5c except for the sequences of catalytic polymers (synthetase efficiency = 0.0005, ligase efficiency = 0.2). Error bars show the 95% Clopper-Pearson confidence intervals. The bracketed number below a column indicates the average count of catalytic sequences or their reverse complements per surviving simulation.** a** Diffusivity is scaled by polymer size (e.g., the ratio of the diffusivity of 8-mers to that of 10-mers equals (10/8)^1/2^). **b** Diffusivity is not scaled by polymer size. **(i)** Synthetase: ABAABABB, ligase: BBAAABAB, 90 replicates. **(ii)** Synthetase: ABAABABB, ligase: ABBAAABABB, 120 replicates. **(iii)** Synthetase: BABAABABBA, ligase: BBAAABAB, 120 replicates. **(iv)** Synthetase: BABAABABBA, ligase: ABBAAABABB, 120 replicates. **(v)** Synthetase: ABAABABB, ligase: ABBAAABABB, 180 replicates. **(vi)** Synthetase: BABAABABBA, ligase: BBAAABAB, 180 replicates. **(vii)** Synthetase: BABAABABBA, ligase: ABBAAABABB, 180 replicates. For (a), apart from columns (ii) and (iii), all other pairs of columns have significantly different survival probabilities. For (b), apart from columns (v) and (vi), all other pairs of columns have significantly different survival probabilities

To test whether our model can replicate the expectation that cooperative systems of longer catalysts are less viable, we used the combination of low-efficiency synthetase and high-efficiency ligase as a reference (Fig. 5 column (c)) and investigated the effect of increasing the functional polymer length to 10. Since our model assumes that larger polymers move slower and, thus, have lower collision frequency (and, thus, a lower effective catalytic efficiency), we were concerned that it would be unclear whether reductions in the probability of surviving for longer polymers were due to the challenges of reliably copying long sequences or their reduced catalytic efficiency. Therefore, we conducted the analyses both with the standard model, where diffusivity is scaled by polymer size (Fig. 6a), and with a modified model that assigns the same diffusivity to 8-mer and 10-mer catalysts (Fig. 6b). In both cases, the combination of 8-mer synthetase and 10-mer ligase (Fig. 6 column (ii)(v)) or 10-mer synthetase and 8-mer ligase (Fig. 6 column (iii)(vi)) have similar viabilities that are lower than the viability of combinations of 8-mer catalysts (Fig. 6 column (i)) and higher than combinations of 10-mer catalysts (Fig. 6 column (iv)(vii)). These results show that holding other properties similar, a system relying on longer catalytic polymers has lower viability. It is worth noting, however, that a higher catalytic efficiency can compensate for the cost of longer polymers, as shown by comparing Figs. 6 column (b) and 5 column (a).

### Independent Mutation Inhibitors can Improve Fidelity of Replication and Persistence of Cooperative Polymer Systems

After validating that our model can replicate key findings of previous studies, we used the model to explore how mutation suppression might emerge and evolve in cooperative polymers systems. It has long been appreciated that early template-guided replication mechanisms in the prebiotic world likely had high error rates (Eigen 1971; Eigen and Schuster 1977, 1978a, b). As a result, it is generally believed that some kind of proofreading would be needed to access the polymer space beyond Eigen’s length threshold. However, paradoxically, adding a proofreading function to a polymerase (or ligase) ribozyme would likely require adding additional functional motifs, and thus additional length, to the molecule. One resolution to this paradox could be the appearance of an independent mutation-inhibiting catalyst that is integrated into an ecosystem of cooperators in such a way as to allow longer polymers to be reliably replicated. This might allow for progressively longer catalysts, perhaps allowing for the eventual emergence of proofreading replicases.

Modeling individual mutation-inhibiting polymers requires that, when the first copy of such a genetic-catalytic polymer arises, its impact on the mutation rate is low until it achieves a higher concentration in the ecosystem. Our model incorporates this factor by tying mutation rate to the count of mutation inhibitors (see Sects. “Methods” and “A Simplified Spatial Model to Efficiently Simulate Prebiotic Genetic-Catalytic Polymers”), which allowed us to explore the effect of individual mutation inhibitors arising alongside pairs of synthetases and ligases. To our knowledge, this is the first exploration of the effects of independent mutation inhibitors in templating polymer systems.

The first set of analyses were similar to the cases seen in Figs. 5 column (d) and 6 column (d), except that we raised the efficiency of the 10-mer synthetase to 0.005 and simulations were initiated with the first reactor containing not only the ligase and synthetase but also a specific 8-mer sequence (ABBABBAA), which was defined as a mutation inhibitor, and its reverse complement. We then explored intrinsic wildcard-production probabilities (*P*_int_) from 0.001 to 0.1 (as contrasted with a rate of 10^–6^ in prior simulations).

**Fig. 7:**
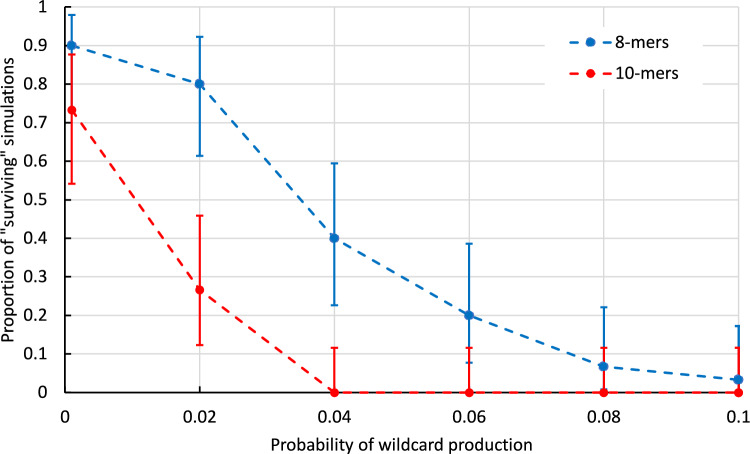
Interaction between mutation propensity and catalyst length influences viabilities of polymer systems. Error bars show the 95% Clopper-Pearson confidence intervals. Probability of wildcard production per Phase 1 varies between 0.001 and 0.1. All data points have 30 replicate simulations. Blue series: Synthetase: ABAABABB, ligase: BBAAABAB. Red series: Synthetase: BABAABABBA, ligase: ABBAAABABB. Except as stated, model parameters were the same as Fig. 5d

Figure 7 confirms that the viability of a polymer system is negatively correlated with the intrinsic wildcard-production probability and, thus, mutation rate. Moreover, a system relying on catalytic 10-mers is more sensitive to mutations than a system relying on catalytic 8-mers. It is noteworthy, however, that systems can persist even with an intrinsic wildcard-production probability as high as 2%, presumably because, if catalytic efficiencies are high enough, polymer systems can synthesize enough correct copies to compensate for the many non-functional descendants that are generated.

To see if a mutation-inhibiting catalyst can rescue the low viability of the 10-mer cooperative system with an intrinsic wildcard-production probability of 0.04 (Fig. 7), we ran another series of simulations over a range of mutation inhibitor efficiencies (*v*) from 0 to 0.9 (see Fig. 1, Sect. “Methods”, and Supplementary Materials). Since the new synthetase-ligase-inhibitor cooperative system had three instead of two functions, viability was redefined as the proportion of simulations where all three functional polymers (and their reverse complements) were present in at least one reactor at the end of simulation.

**Fig. 8:**
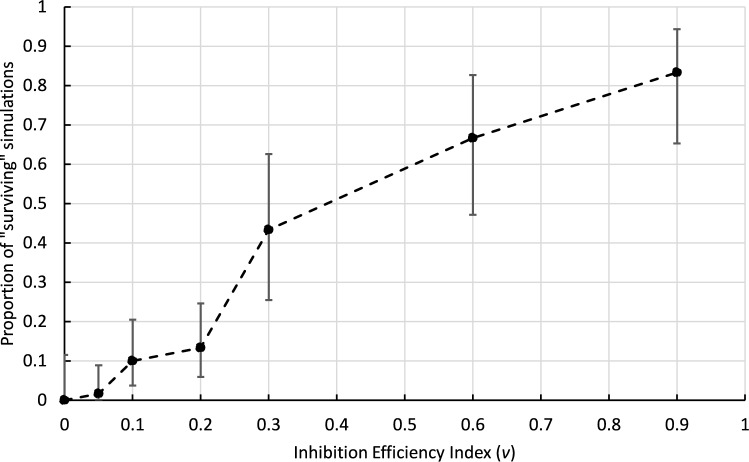
Mutation inhibitor rescues the viability of a cooperative polymer system. Model parameters are the same as the red data point with intrinsic wildcard-production probability being 0.04 in Fig. 7, except that the efficiency of the mutation inhibitor ABBABBAA varied from 0 to 0.9. Error bars show the 95% Clopper-Pearson confidence intervals. For *v* ∈ {0, 0.3, 0.6, 0.9} there are 30 replicate simulations; for *v* ∈ {0.05, 0.1, 0.2} there are 60 replicate simulations. Viability (vertical axis) is measured by the proportion of simulations where synthetase BABAABABBA, ligase ABBAAABABB, mutation inhibitor ABBABBAA, and their reverse complements are all present in at least one reactor at the end of the simulation

As predicted, the mutation inhibitor greatly increases the viability of the polymer system (Fig. 8). Interestingly, the survival probability when a mutation inhibitor is present with a 10-mer synthetase and ligase (Fig. 8) can equal or exceed that seen when the synthetase and ligase are 8-mers but there is no mutation inhibitor (blue line in Fig. 7).

## Discussion

In this study, we developed an abstract model of prebiotic chemical ecosystems with template-guided replication and used it to explore how the ability to inhibit errors could have emerged. Our model is inspired by real-world processes but uses an abstract framework to enable exploration of diverse factors that could affect the persistence and evolution of templating catalytic polymer systems. Such abstraction helps avoid overfitting extant biology while also circumventing the combinatorial explosion arising from more conventional and realistic models.

Our study replicated prior findings that a spatial structure conducive to multilevel selection facilitates the persistence of cooperative polymer systems (Szabó et al. 2002; Takeuchi and Hogeweg 2009; Shay et al. 2015; Czárán et al. 2015; Kim and Higgs 2016; Matsumura et al. 2016; Mizuuchi and Ichihashi 2018). The study goes beyond prior results by demonstrating that, given such a spatial structure, a genetic-catalytic polymer capable of inhibiting mutations in a dosage-dependent manner can survive and become enriched even when it emerges in a system with a high mutation rate.

Our results suggest a pathway by which the genetic system of modern life could have emerged and overcome Eigen’s error threshold paradox (Eigen 1971; Eigen and Schuster 1977, 1978a, b). Specifically, it seems plausible that ecosystems of short polymers catalyzing diverse, complementary functions, such as the synthesis of activated nucleotides (metabolic catalysts, synthetases, etc.), RNA replication (e.g., polymerases, ligases), and mutation inhibition, could have emerged in conditions conducive to multilevel selection. In such settings, improvements in the production of monomers, increases in the efficiency of their polymerization, and gains in replication fidelity could gradually accumulate and allow progressively longer (and potentially more efficient) catalytic polymers to become viable. This stepwise evolution could entail new catalysts that fulfill novel functions (e.g., making membranes, or enabling more efficient metabolism) as well as ones that fulfill pre-existing functions, but with greater efficiency. Among other catalytic functions, it is easy to imagine that such progressive complexification could result in the eventual emergence of proofreading polymerases.

The approach we used here was to presume the existence of simple catalytic polymers and investigate conditions conducive to their survival in an environment that almost never generates polymers without catalysts. While useful, this approach does not address the initial source of genetic diversity. One possibility is that non-catalytic synthesis and ligation was sufficient for the shortest functional polymers, and their reverse-complements, to emerge by chance. Alternatively, we can imagine two classes of environments connected by diffusion: one spontaneously producing abundant polymers and another where the production of polymers depends heavily on catalytic polymers. In such a situation, polymer dispersal from the former to the latter class of environments would set up the kinds of situations modelled here, where catalytic polymers appear stochastically but can only persist when they cooperate effectively with other polymers.

Our results, especially Sect. “Independent Mutation Inhibitors can Improve Fidelity of Replication and Persistence of Cooperative Polymer Systems”, demonstrate that this model is flexible, and can be extended to explore diverse intrinsic and extrinsic factors. A few possible extensions are worth mentioning. We used a 1D circular array of reactors, but many real-world settings, such as mineral surfaces, porous rocks, or hydrothermal fields/vents would be better represented by 2D (or even 3D) reactor arrays. It would be informative, therefore, to examine how reactor-array geometry alters the effects of multilevel selection and how this, in turn, influences the fate of polymer ecosystems.

In this study, we only considered two types of directional monomers that match in a reverse-complementary manner. It would be easy enough to consider reverse-complementary systems with four bases, analogous to RNA, or to consider potential alternatives mode of templating. For example, one could readily consider models in which monomers and polymers lack directionality (analogous to 3’-5’ in RNA), models in which base-pairing is directly complementary (e.g., ABB matching BAA instead of AAB), or even models where pairing happens between monomers of the same type (e.g., A matching A, B matching B), etc. To simulate these alternative scenarios, one could simply make changes to the adsorption phase and the rules determining reactive borders (Fig. 1).

We only considered three catalytic functions in this work: synthesis of activated monomers, formation of polymer backbones, and mutation inhibition. Many more biochemical functions are conceivable. For example, the reaction phase (Fig. 1) could be modified to make the synthesis of free activated monomers consist of additional steps, better mimicking complex metabolic pathways. Similarly, while it would require more complex tracking of adsorbed molecular complexes, we could allow catalysts to bind to substrates so as to allow phenomena such as processive polymerization. Alternatively, we could allow polymers to confer structural rather than catalytic functions, for example changing surface properties in such a way as to reduce the rate of loss by dilution.

The approach taken here, of seeding ecosystems with catalytic polymers, is sufficient to explore catalyst persistence but has limited utility for exploring how new functional sequences arise over time. To simulate open-ended adaptive evolution, it would be necessary to add a module for automatically assigning catalytic functions (of diverse kinds) and catalytic efficiencies to newly emerged sequences. Using such a module, one could potentially study the process by which short, inefficient catalysts arise and are gradually replaced by new, more efficient (and perhaps longer) polymers. Such an approach should make it possible to explore long-term complexification in ecosystems of cooperating polymers, all driven by multilevel selection. Such an extension would greatly advance our understanding of the origins of life by better explaining the co-emergence and co-complexification of metabolisms and genetic information processing systems.

## Methods

### Detailed Explanation of the Four Phases

In our model, molecules can be adsorbed to the bottom surface of a reactor; an adsorbed molecule may or may not have directionality along the horizontal dimension. Polymers are built from activated monomers A and B that have directionality and the capacity for reverse-complementary base-pairing, analogous to nucleotide triphosphates. Synthetase polymers catalyze the reversible reactions E + F ⇌ A + W and E + G ⇌ B + W, which synthesize activated free monomers A and B from energetic food (E) and structural foods (F, G). Activated monomers (A, B) and structural food (F, G) are directional, meaning that the two borders of sites that they occupy have different possible reactions. Energetic food and waste, in contrast, are non-directional. Synthetase activity generates the building blocks of catalytic and genetic polymers, as in metabolism.

To enable mutation, we allow that during Phase 1, A and B can stochastically acquire a “wildcard” status, M or N, respectively, which allows them to pair equally well with any monomer types. Such a mode of mutation resembles chemical changes to nucleic acids (Ryu et al. 2018; Licht et al. 2019; Srinivasan et al. 2021), such as certain methylation or deamination reactions, which have the potential to be repaired. Nevertheless, when modelling mutation inhibition, the underlying physicochemical mechanisms are not that important: as long as the probability of A or B to mutate to a wildcard has a reachable upper limit representing the intrinsic propensity to mutate, an unreachable lower limit at 0 representing the fact that mutation is not avoidable, and is negatively correlated to the quantity of mutation inhibitors in local environment, the model can generate clues about how mutation inhibitors might emerge, survive, and evolve in a prebiotic environment.

Ligase activity provides the basis for genetic encoding insofar as ligase catalysis is sensitive to base-pairing with a template sequence, meaning that there is a tendency for genetic information to pass from a template to a complementary strand (Tkachenko and Maslov 2018). In our model, a ligase can link monomers regardless of whether they are free or incorporated in a polymer; for example, a ligase would act on reactions such as ABAA + B ⇌ ABAAB and ABA + AB ⇌ ABAAB.

To simulate templated-guided synthesis of polymers, our model considers a reactor of which the bottom surface is narrow enough such that it can be viewed as a quasi-1D line. Alternatively, such a reactor may be viewed as a discrete projection of a 2D surface to a 1D line, as long as the 2D surface is large enough such that polymers directly adsorbed to the surface (see below) are unlikely to form crosses. This linear bottom surface can adsorb two layers of molecules – the first layer which is directly adsorbed to the surface and the second layer which is adsorbed to first-layer monomers or polymers by base-paring (like the game Tetris®), leaving other molecules (if any) moving around in the solution above these layers. The model cycles through four phases: monomer modification, adsorption, reaction, and resuspension (Fig. 1). Since synthetase-catalyzed and ligase-catalyzed reactions only occur at the borders between adsorbed molecules, the possibility of a reaction occurring depends only on the occupants of adjacent surface sites (Fig. 1c). As a result, during the reaction phase, the model simply needs to track and update the states of the borders where reactions may occur, meaning that it is not necessary to track individual polymer sequences.

In Phase 1 (monomer modification), free and incorporated monomers were first assigned an intrinsic probability* P*_int_ of becoming wildcards. However, the overall probability *P*_wild_ of normal monomers becoming wildcards also depends on the action of a catalytic mutation inhibitor species. The relation is accounted for by a function *P*_keep_(*v*, *x*) where *v* and *x* are the efficiency and quantity of the mutation inhibitor species, respectively. By letting *P*_wild_ = *P*_int_*P*_keep_(*v*, *x*) and assuming that *P*_keep_(*v*, *x*) = (*1*–*v*)^*x*^ with *v* ∈ (0, 1), the model ensures that the probability of monomers entering Phase 2 as wildcards is negatively correlated to the efficiency and quantity of mutation inhibitors while never reaching 0. Note that *P*_wild_ does not directly equal the mutation rate (in mutations per monomer per cycle), because a wildcard does not necessarily lead to mismatch (e.g., a wildcard of A happens to match a normal B) and a mismatch does not necessarily lead to a mutation (e.g., the first-layer template happens to be a single free monomer). Nonetheless, the realized mutation rate should relate positively (and monotonically) to the wildcard frequency and, thus, negatively to the quantity and efficiency of mutation inhibitors. The same rationale can also be used to deal with multiple mutation inhibitor species with different quantities and efficiencies (see Supplementary Materials).

In Phase 2 (adsorption), polymers, free monomers, energetic and structural foods, and waste are adsorbed to the surface until no more adsorption can occur (Fig. 1b). Any free monomers or polymers that are not adsorbed in the first layer can be adsorbed onto a second layer, similar to the way that a Tetris® block can fall on another block (Fig. 1b). The second-layer adsorption follows reverse-complementary base-pairing rules and continues until no more adsorption can occur. After this phase, molecules or incorporated monomers that occupy adjacent sites determine whether the borders between these sites are “reactive borders” (Fig. 1c), where a bond may be formed or broken during the reaction phase. The spatial structure of the surface within a compartment is the key to avoiding a combinatorial explosion, because the spatial structure is a prerequisite for defining reactive borders and it is the surface size (rather than the number and size of molecules) that determines of the maximum number of reactive borders. This adsorption-based catalysis is, however, consistent with prior empirical and theoretical evidence that the formation of oligomers, the phenomenon of templating, and nucleotide polymerization can result from wet-dry cycling of nucleotides (Olasagasti et al. 2011; Higgs 2016; Hassenkam and Deamer 2022; Song et al. 2024).

In Phase 3 (reaction), catalytic polymers that were not adsorbed are allowed to randomly collide with borders, and all wildcards now act as normal monomers (Fig. 1c). Whenever a catalytic polymer (e.g., ligase) collides with a reactive border, it has a probability, its *catalytic efficiency*, of causing a reaction to occur. For example, a reaction might entail two unconnected monomers becoming connected (Fig. 1c, black triangle), a polymer being split (Fig. 1c, empty triangle), or a structural food reacting with an energetic food to generate a free monomer and waste (Fig. 1c, black diamond). For a catalytic polymer, the number of collisions with borders within a reaction phase follows a Poisson distribution and is negatively correlated to the polymer mass (reflecting the slower diffusion of larger molecules). Spontaneous reactions can be implemented by assuming that natural catalysts are present at some baseline concentration.

The number of reaction events that occur in the reaction phase is mainly determined by the number of catalytic polymers and the number of reactive borders. Under this approach there is no need to re-calculate reaction propensities within an iteration since the number of catalysts and reactive borders does not change and there are only two general types of reactive border: (i) the borders between non-directional molecules (E, W) and properly oriented directional foods (F, G) or properly oriented free monomers (A, B) in the first layer, which may undergo the conversion of energetic and structural foods into free monomers and waste or the reverse reaction, and (ii) the borders between similarly-oriented monomers in the second layer, which may undergo the formation or breakage of bonds between monomers. In other words, the synthesis of activated monomers and its reverse reaction only occur when E, W, F, G, free A, and free B are properly oriented in the first layer and, similarly, ligation (and its reverse reaction) only occurs when polymers and free monomers that are properly oriented in the second layer. The strategy of only tracking reactive borders circumvents the combinatorial explosion problem, and can be thought of as a case where catalysts are shortsighted, and only care about whether a border on the surface can be attacked, regardless of what molecules are consumed or produced after a bond is formed or broken. As a result, reactions that are catalyzed by the same type of catalysts are not independently tracked but lumped together and only the sites next to reactive borders have their statuses tracked, which dramatically decreases the computational load.

In Phase 4 (resuspension), all adsorbed molecules are released (Fig. 1d), returning the system to the original configuration with a different set of molecules. Given that at least some synthetase and ligase polymers exist at the beginning, the iteration of wildcard modification, adsorption, reaction, and resuspension for multiple cycles is conceptually similar to iterating the steps of annealing, extension, and denaturation in PCR. Multiple such cycles form a generation (Fig. 1).

More details of the model can be found in Supplementary Materials.

## Supplementary Information

Below is the link to the electronic supplementary material.

Supplementary file1 (DOCX)

Supplementary file1 (PDF)
